# Fibrin-Targeted Nanoparticles for Finding, Visualizing and Characterizing Blood Clots in Acute Ischemic Stroke

**DOI:** 10.3390/pharmaceutics14102156

**Published:** 2022-10-10

**Authors:** María Luz Alonso-Alonso, María Pérez-Mato, Ana Sampedro-Viana, Clara Correa-Paz, Paulo Ávila-Gómez, Tomás Sobrino, Francisco Campos, José Castillo, Ramón Iglesias-Rey, Pablo Hervella

**Affiliations:** 1 Neuroimaging and Biotechnology Laboratory (NOBEL), Clinical Neurosciences Research Laboratory (LINC), Health Research Institute of Santiago de Compostela (IDIS), 15706 Santiago de Compostela, Spain; 2 Neurological Sciences and Cerebrovascular Research Laboratory, Department of Neurology and Stroke Center, Neuroscience Area of IdiPAZ Health Research Institute, La Paz University Hospital, Universidad Autónoma de Madrid, 28046 Madrid, Spain; 3 Translational Stroke Laboratory (TREAT), Clinical Neurosciences Research Laboratory (LINC), Health Research Institute of Santiago de Compostela (IDIS), 15706 Santiago de Compostela, Spain; 4 NeuroAging Laboratory Group (NEURAL), Clinical Neurosciences Research Laboratory (LINC), Health Research Institute of Santiago de Compostela (IDIS), 15706 Santiago de Compostela, Spain

**Keywords:** fibrin, MRI, nanoparticles, stroke, targeting, thrombolysis, rtPA

## Abstract

Recanalization of the occluded artery is the gold standard treatment for acute ischemic stroke, which includes enzymatic fibrinolytic treatment with the use of recombinant tissue plasminogen activators (rtPAs) to disrupt the occluding clot, the use of mechanical thrombectomy to physically remove the clot, or a combination of both. Fibrin is one of the main components of blood clots causing ischemic stroke and is the target of rtPA upon activation of plasminogen in the clot. In addition, fibrin content also influences the efficacy of mechanical thrombectomy. Current imaging methods can successfully identify occlusions in large vessels; however, there is still a need for contrast agents capable of visualizing small thrombi in ischemic stroke patients. In this work, we describe the synthesis and the in vitro characterization of a new diagnostic nanoparticle, as well as the in vivo evaluation in an animal model of thromboembolic stroke. Gd-labeled KCREKA peptides were synthesized and attached onto the surface of PEGylated superparamagnetic nanoparticles. Magnetic resonance imaging (MRI) of blood clots was performed in vitro and in vivo in animal models of thromboembolic stroke. KCREKA-NPs were synthesized by attaching the peptide to the amino (N) termini of the PEG-NPs. The sizes of the nanoparticles, measured via DLS, were similar for both KCREKA-NPs and PEG-NPs (23 ± 4 nm, PDI = 0.11 and 25 ± 8 nm, PDI = 0.24, respectively). In the same line, r_2_ relaxivities were also similar for the nanoparticles (149 ± 2 mM Fe s^−1^ and 151 ± 5 mM Fe s^−1^), whereas the r_1_ relaxivity was higher for KCREKA-NPs (1.68 ± 0.29 mM Fe s^−1^ vs. 0.69 ± 0.3 mM Fe s^−1^). In vitro studies showed that blood clots with low coagulation times were disrupted by rtPA, whereas aged clots were almost insensitive to the presence of rtPA. MRI in vitro studies showed a sharp decrease in the T_1_ × T_2_ signals measured for aged clots incubated with KCREKA-NPs compared with fresh clots (47% [22, 80] to 26% [15, 51]). Furthermore, the control blood showed a higher value of the T_1_ × T_2_ signal (39% [20, 61]), being the blood clots with low coagulation times the samples with the lowest values measured by MRI. In vivo studies showed a significant T_1_ × T_2_ signal loss in the clot region of 24% after i.v. injection of KCREKA-NPs. The thrombus age (2.5% ± 6.1% vs. 81.3% ± 19.8%, *p* < 0.01) confirmed our ability to identify in vivo fresh blood clots. In this study, we developed and tested a dual MRI nanoparticle, acting as T_1_ and T_2_ contrast agents in MRI analyses. The developed KCREKA-NPs showed affinity for the fibrin content of blood clots, and the MRI signals provided by the nanoparticles showed significant differences depending on the clot age. The developed KCREKA-NPs could be used as a tool to predict the efficacy of a recanalization treatment and improve the triage of ischemic stroke patients.

## 1. Introduction

Ischemic strokes are caused by a thrombotic or an embolic occlusion of an artery in the brain, resulting in neuronal cell death via several mechanisms, known as the ischemic cascade [1]. Recanalization of the occluded artery is the gold standard treatment for acute ischemic stroke, which includes enzymatic fibrinolytic treatment with the use of recombinant tissue plasminogen activators (rtPAs) to disrupt the occluding clot, the use of mechanical thrombectomy to physically remove the clot, or a combination of both [2]. The functional outcomes of ischemic stroke patients are associated with success in the rapid recanalization of the occluded vessels [3]. Although there is a proven benefit in the functional outcomes for patients with effective rtPA-mediated recanalization, several drawbacks are associated with the fibrinolytic therapy with rtPA, such as the low recanalization rate (less than 30%) [4,5] and the harmful hemorrhagic and neurotoxic effects of rtPA, which are particularly present in patients with ineffective recanalization [6]. In addition, the administration of rtPA was limited to patients arriving at the hospital within 4.5 h from the stroke onset, since this drug is highly ineffective and potentially dangerous after that time [7]. In this regard, the ability to predict which patients can be successfully treated with rtPA would improve the management of ischemic stroke.

Mechanical thrombectomy is currently the most successful treatment for the recanalization of large-vessel occlusions in ischemic stroke patients. The successful removal of a blood clot via mechanical thrombectomy is associated with the mechanical properties of the blood clot, which are also dependent on the fibrin content of the clot [8]. In this regard, clinical studies have demonstrated that the administration of rtPA before mechanical thrombectomy resulted in a reduction in the size of the retrieved clot and in a modification of its components, but not in a better recanalization rate [9,10]. Conversely, recent findings have indicated that the administration of rtPA after a successful mechanical thrombectomy resulted in a better neurological outcome in stroke patients [11]. This improvement could be attributed to the fibrinolytic effect of rtPA on the small, persistent thrombi present in the microcirculation at the end of the thrombectomy. These thrombi affect the viability of the neighboring neurons, and rtPA could be potentially used to treat them based on their fibrin content [12]. In this work, we describe a new tool to predict the fibrinolysis rate of rtPA based on the magnetic resonance imaging (MRI) signal provided by nanoparticles (NP) sensitive to the fibrin content of the occluding clots.

Fibrin is one of the main components of blood clots causing ischemic stroke and is the target of rtPA upon the activation of plasminogen in the clot [13]. Consequently, the fibrin content in clots strongly influences the activity of rtPA [14,15]. Fibrin-rich clots (in cardioembolic strokes) are more likely to be disrupted by rtPA, whereas clots rich in platelets (atherothrombotic strokes) are less sensitive to rtPA [16]. In the clinical practice, this is reflected in rtPA recanalization rates, depending on the stroke subtype [17,18]. For instance, large atherothrombotic strokes, caused by the disruption of an atheroma plaque that is rich in platelets, show the lowest recanalization rates [19]. In addition, fibrin content also influences the efficacy of mechanical thrombectomy, which is reflected by a higher recanalization rate in patients with fibrin-rich clots analyzed via clot histology [8]. Thus, direct visualization of the fibrin content in a thrombus may be beneficial for both diagnosis and recanalization therapy.

Current imaging methods can successfully identify occlusions in large vessels [20,21]; however, there is still a need for contrast agents capable of visualizing small thrombi in ischemic stroke patients. Several fibrin-binding peptides labeled with Gd as magnetic resonance imaging (MRI) contrast agents have been developed for thrombus detection [22,23,24]. For instance, the peptide EP-2104R showed promising results in clinical trials in the detection of non-visible thrombi through pre-contrast imaging [25,26]. Furthermore, based on fibrin-binding peptides, nanoparticles targeted at clots were developed for thrombus imaging [27,28], taking advantage of the higher contrast provided by nanoparticles compared with labeled peptides. In this work, we propose to use a combination of both strategies, using nanoparticles as MRI contrast agents decorated with fibrin-targeted peptides. The main difference between these and other fibrin-targeted nanoparticles is the presence of Gd in the peptide, providing the nanoparticles with an additional MRI imaging tool that is sensitive to the fibrin content in the blood clot. Since nanoparticles targeted towards fibrin by means of unlabeled peptides can be used for detecting blood clots [29], we hypothesized that peptides labeled with Gd attached to the nanoparticle surface could provide an additional MRI signal that may be sensitive to the fibrin content in blood clots.

In this work, we describe the synthesis and the in vitro characterization of a new diagnostic nanoparticle, as well as the in vivo evaluation in an animal model of thromboembolic stroke, in which the beneficial effect of rtPA-induced reperfusion and hemorrhagic transformation associated with delayed administration are similar to those occurring in humans. The developed nanoparticles could represent a valuable tool for the detection and diagnosis of small blood clots in ischemic stroke patients.

## 2. Materials and Methods

### 2.1. Materials

The peptide with the sequence NH_2_-Lys-Cys-Arg-Glu-FmocLys-Ala-COOH (KCREK*A) was purchased from Biomatik Corporation. (Ontario, Canada); Polyethylene glycol (PEG) diamine (2000, 3000, 6000, and 10,000 Da) was purchased from Polysciences (Warrington, PA, USA); 1,4,7,10-tetraazacyclododecane-1,4,7,10-tetraacetic acid mono-N-hydroxysuccinimide ester (DOTA-NHS) was purchased from Macrocyclics (Plano, TX, USA); and recombinant tissue plasminogen activator (rtPA, Alteplase, Actilyse^TM^), was purchased form Boehringer Ingelheim (Ingelheim, Germany). All the other reagents were purchased from Merck (Damstadt, Germany).

### 2.2. Synthesis of Gd-Labeled Peptides

The peptide with the sequence KCREK*A (NH_2_-Lys-Cys-Arg-Glu-FmocLys-Ala-COOH) was selected as the starting point based on the fibrin-binding peptide CREKA (Cys-Arg-Glu-Lys-Ala). The peptide (9.6 mg, 10.0 µmol) was first dissolved and oxidized in water and dimethyl sulfoxide (10% *v*/*v*) under vigorous stirring overnight. The resulting dimer was conjugated to DOTA by reacting with a 3-fold molar excess of DOTA-NHS (22.8 mg, 30 µmol) in carbonate buffer at pH = 9 for 4 h. Finally, pH was adjusted to 5 with 2-(*N*-morpholino) ethanesulfonic acid (MES) buffer and GdCl_3_ (16 mg, 40 µmol) was added to the solution and stirred overnight. The resulting peptide was then stored at −20 °C. The reaction was monitored using a high-performance liquid chromatography (HPLC) system (Agilent 1200 series) with a C18 analytical column (Kromasil 100-5-C18, 250 mm, pore size 5 mm). The mobile phase used a gradient from 100% solvent A (acetonitrile) to 30% A and 70% of solvent B (0.1% trifluoroacetic acid in water) at 25 min and 100% solvent B at 30 min at a flow rate of 1 mL/min. The sample injection was 20 µL and the detection wavelength was 220 nm (Figure S1A). Furthermore, the final product and all intermediates were measured via Matrix-assisted laser desorption/ionization (MALDI) mass spectrometry in a 4800 MALDI-TOF/TOF system (Sciex).

### 2.3. Synthesis of KCREKA-Targeted Nanoparticles (KCREKA-NPs)

KCREKA-NPs were synthesized from polyacrylic acid (PAA)-coated nanoparticles (PAA-NPs) synthesized following the coprecipitation method in the presence of PAA [30]. Briefly, PAA (600 mg, 286 µmol) was dissolved in degasified water (100 mL) in a round-bottom flask, then NH_4_OH (6 mL, 43 mmol) was added, and the solution was heated at 70 °C and stirred under N_2_. FeCl_3_ (300 mg, 1.8 mmol) and FeCl_2_ (120 mg, 0.95 mmol) were dissolved in 50 mL HCl 0.01 M and the mixture was then added dropwise to the PAA solution under vigorous stirring for 30 min under N_2_. The resulting black suspension of PAA-coated nanoparticles (PAA-NP) was then heated under vacuum conditions to remove the excess of NH_4_OH and washed five times with MilliQ water using an Amicon ultracentrifugal filter (Millipore, Burlington, MA, USA) with a molecular-weight cutoff of 100 kDa.

PEG-NPs were synthesized from PAA-NPs (1 mL, 190 µmol Fe_3_O_4_) by adding NHS (200 µmol), EDC (200 µmol), and diamino PEG (20 µmol) in carbonate buffer (pH = 9) for 2 h [31]. The mixture was washed five times with water to remove the unbounded PEG using an Amicon ultracentrifugal filter (Millipore) with a molecular-weight cutoff of 100 kDa.

Gd-PEG-NPs were prepared from PEG-NPs (0.5 mL, 23.7 µmol Fe_3_O_4_, 2.5 µmol PEG) by adding DOTA-NHS (20 µmol) to the suspension and stirring for 2 h in carbonate buffer (ph = 9). The mixture was washed via ultrafiltration as described above, and then a solution of GdCl_3_ (50 µmol) in MES buffer (pH = 5) was added to the NP. The mixture was stirred overnight at room temperature and the excess Gd was removed via ultracentrifugation as described above.

KCREKA-NPs were synthesized starting with the Gd-modified dimer KCREKA peptide described above. The peptide (20 mg, 5 µmol) was dissolved in MES buffer and reacted with NHS (7 µmol) and EDC (7 µmol) for 15 min; then, PEG-NP (47.4 µmol Fe_3_O_4_, 5 µmol PEG 6 kDa) was added to the mixture, its pH was adjusted to 9, and it was stirred for 4 h at room temperature. The resulting KCREKA-NPs were washed via ultracentrifugation and then transferred to a piperidine solution (50% *v*/*v*) and stirred overnight to remove the F-Moc protecting group present in the peptide. The final KCREKA-NPs were then washed five times with water using an Amicon ultracentrifugal filter (Millipore) with a molecular-weight cutoff of 100 kDa.

The peptide concentration in the resulting filtrate was measured via HPLC as described above (Figure S1B), and the percentage of peptide conjugated to nanoparticles was calculated using the formula:

(1) $$Cojugation\;rate=\frac{Peptide_{Total}-Peptide_{Filtrate}}{Peptide_{Total}}\times 100$$

The Fe and Gd concentrations of each nanoparticle were measured via mass spectrometry with inductively coupled plasma after dissolving the nanoparticles in HNO_3_/HCl. Particle size and polydispersity were measured via dynamic light scattering in a Nanosizer 2000 (Malvern Instruments, Malvern, UK). Transmission electron microscopy (TEM) was carried out without staining in a JEOL JEM 2010 electron microscope.

### 2.4. Magnetic Resonance Imaging (MRI) Experiments

All studies were conducted on a Bruker Biospec 9.4 T MR (Bruker, Billerica, MA, USA) scanner (horizontal bore magnet with 12 cm wide Bruker BioSpin) equipped with actively shielded gradients (440 mT m^−1^). For the in vitro acquisition data, a radiofrequency resonator was used as transmitter/receiver (with a quadrature volume coil of 7 cm in diameter). Animals were imaged with a combination of a linear birdcage resonator (7 cm in diameter) for signal transmission and a 2 × 2 surface coil array for signal detection, positioned over the head of the animal, which was fixed with a teeth bar, earplugs, and adhesive tape. Transmission and reception coils were actively decoupled from each other.

### 2.5. Measurements of Nanoparticles T_1_ and T_2_ Relaxivities

The T_1_ relaxivity (r_1_) and T_2_ relaxivity (r_2_) of each nanoparticle, as a measure of its value as an MRI contrast agent, was evaluated at 9.4 T in an agar (1.6% *w*/*v*) mold with multiple wells, following a procedure described elsewhere [32]. Nanoparticles were first diluted in water to obtain Fe concentrations ranging from 0 to 0.5 mM, then mixed with liquid agar (70 °C) and placed in the solid agar mold. The mold was then sealed with liquid agar and cooled to room temperature. T_2_-weighted images (T_2_-wis) were acquired using the multi-slice multi-spin-echo (MSME) sequence with 11.32 ms of echo time (ET), 3 s of repetition time (RT), 16 echoes with 11.32 ms echo spacing (ES), 50 kHz spectral bandwidth (SB), a flip angle (FA) of 90°, 14 slices of 1 mm, and 1 average. T_2_-wis were obtained with a field of view (FOV) of 7.5 × 7.5 cm^2^ (with saturation bands to suppress signals outside this FOV) and a matrix size of 300 × 300, giving an in-plane resolution of 250 μm/pixel and implemented without fat suppression. T_1_-weighted images (T_1_-wis) were acquired using a rapid acquisition with a relaxation enhancement-variable (RARE-VTR) sequence with an ET = 17.48 ms, RT = 1 s, NA = 1, number of repetitions (NR) = 1, SW = 50 kHz, rare factor (RF) = 4, 6 T_1_ experiments (RT = 900, 2000, 3500, 5000, 7000, 11,000 ms), and with the same T_2_ geometry.

### 2.6. In Vitro Fibrinolysis Assay

The fibrinolysis studies were carried out following the standard protocols adapted to this study [33]. Briefly, fresh blood was obtained from healthy rats (*n* = 3) and 100 µL were placed in Eppendorf tubes. The blood was incubated at 37 °C for 1, 2, 4, 6, and 24 h for coagulation and then the samples were washed 2–3 times with PBS, avoiding the disaggregation of the clot. Then, the PBS was removed, leaving the clot in the tube. The samples were weighted and then 50 µL of rat serum and 100 µL of rtPA (100 µg/mL) in PBS were added to the samples. As controls, samples were also incubated with PBS without rtPA at the same time points and conditions as the study group. The samples were then incubated at 37 °C for 90 min under mild agitation and then centrifuged at 5000 rcf for 10 min. The supernatants were then removed, and the clots were dried at 37 °C overnight. The dry samples were weighted and then 1 mL of MilliQ water was added to lyse the remaining erythrocytes. Samples were then centrifuged and the absorbance of the supernatant was measured in a plate reader (Biotek Instruments, Winooski, VT, USA) at 560 nm. The percentage of clot lysis was then calculated based on the value of the absorbance adjusted by the clot weight. 

### 2.7. In Vitro Clot MRI

Fresh blood was obtained from healthy rats (*n* = 3) and placed in a custom-made agar phantom. The blood was placed in an incubator at 37 °C for 1, 4, 24, and 48 h for clot formation. Blood with anticoagulant (ethylenediamintetraacetic acid, EDTA), incubated for 1 h, was used as a control. After clot formation, nanoparticles were added to the samples and incubated for 1 h, then the phantom was washed with PBS to remove the unbound nanoparticles, sealed with agar, and placed in the MR scan system for analysis (T_1_-wi, T_2_-wi), as described above in the MRI protocols section.

### 2.8. Experimental Animals

All experimental animal procedures were conducted under the procedure number 15011/2021/003, approved by the Animal Care Committee, according to European Union Rules and the Spanish regulation (86/609/CEE, 2003/65/CE, 2010/63/EU, RD1201/2005 and RD53/2013, RD 53/2013). Twenty-three Sprague–Dawley rats with weights between 200 and 250 g were used for in vivo studies. Animals were kept in a controlled environment at 22 °C ± 1 °C and 60% ± 5% humidity, with 12:12 h light:darkness cycles and were fed ad libitum with standard diet pellets and tap water. All surgical procedures and MRI studies were conducted under sevoflurane (Abbott Laboratories, IL, USA) anesthesia (3%–4%) using a carrier 65:35 gas mixture of N_2_O:O_2_. Animals were sacrificed within the 24 h after the MR examination.

### 2.9. In Vivo Clot MRI

Two in vivo MRI experiments were performed (see the protocol diagram in Figure S2). The thromboembolic stroke model was produced using a previously described protocol [34,35]. Briefly, an autologous clot (5 mm long × 0.58 mm wide) was introduced through the right external carotid artery up to the intracranial internal carotid artery bifurcation to occlude the middle cerebral artery (MCA) at its origin. Cerebral blood flow was monitored with a Periflux 5000 laser-Doppler system (Perimed AB, Järfälla, Sweeden) by placing the Doppler probe (model 411, Perimed AB) in the parietal bone surface near the sagittal crest, under the temporal muscle. Ten minutes after the artery was occluded (determined by a reduction in the Doppler signal), the anesthetized animals were moved carefully in less than 1 min from the surgical bench to an MR to confirm the basal ischemic lesion with apparent diffusion coefficient (ADC) maps, and angiographic imaging. Baseline imaging studies were performed to detect the thrombus region (T_1_-wi, and T_2_-wi 3D sequences). In the first in vivo imaging experiment (*n* = 3 animals), autologous clots of 2 h incubation time were used to occlude the MCA. After the baseline MR scans were completed, 0.3 mL of KCREKA-NPs (peptide dose 1.2 µmol/kg) was administered as a bolus through the tail vein (intravenous injection (i.v.)). Postcontrast sequences, T_1_-wi, and T_2_-wi 3D were acquired. In the second study, we compared 2 h vs. 24 h incubation clots. These clots were incubated in (i) PBS, (ii) NP, or (iii) KCREKA-NP solutions for 5 min immediately before MCA occlusion to study the changes in the MRI signal due to the clot age (*n* = 3 animal/group for 2 and 24 h clots).

ADC maps were acquired during MCA occlusion from diffusion-weighted images (DWI) using a spin-echo echo-planar imaging sequence (DTI-EPI): ET = 24.89 ms; RT = 4.5 s; SW = 200 kHz; 7 b-values of 0, 300, 600, 900, 1200, 1600, and 2000 s·mm^−2^; FA = 90°; NA = 3; 14 consecutive slices of 1 mm; 24 × 16 mm^2^ FOV (with saturation bands to suppress signals outside this FOV); a matrix size of 96 × 64 (isotropic in-plane resolution of 250 μm/pixel); and implemented with the fat suppression option. Time-of-flight magnetic resonance angiography (TOF-MRA) was obtained using a 3D-Flash sequence with an ET = 2.8 ms, RT = 15 ms, FA = 30°, NA = 2, SW = 98 kHz, 1 slice of 14 mm, 30.72 × 30.72 × 14 mm^3^ FOV (with saturation bands to suppress signals outside this FOV), and a matrix size of 256 × 256 × 58 (resolution of 120 × 120 × 241 μm^3^/pixel), and implemented without the fat suppression option. For 3D-T_1_ we used a flash sequence with an ET = 8 ms, RT = 50 ms, FA = 20°, NA = 1, NR = 1, SW = 277 kHz, 1 slice of 16 mm, 32 × 32 × 16 mm^3^ FOV (with saturation bands to suppress signal outside this FOV), and a matrix size of 320 × 320 × 64 (100 × 100 × 250 μm^3^/pixel), implemented without the fat suppression option, and performed with inferior saturation to null inflowing arterial blood. 3D-RARE T_2_ was obtained with ET = 40 ms, RF = 16, RT = 1800 ms, NA = 1, NR = 1, FA = 90°, SW = 98 kHz, 1 slice of 16 mm, 32 × 32 × 16 mm^3^ FOV (with saturation bands to suppress signal outside this FOV), and a matrix size of 256 × 256 × 32 (resolution of 125 × 125 × 500 μm^3^/pixel), implemented without the fat suppression option, and performed with inferior saturation to null inflowing arterial blood.

### 2.10. Data Analysis and Statistics

Images were processed and their MR angiography, ADC, and T_1_ and T_2_ map results were calculated using FIJI: ImageJ software (Rasband W, NIH, Bethesda, MD, USA). For the in vitro experiments, T_1_ and T_2_ maps were calculated from T_1_- and T_2_-wis for fresh and old clots incubated with KCREKA-NPs and control PEG-NPs. We used a combination of the T_1_ × T_2_ signal with the aim of removing false positives and to further decrease the MRI signals. Regarding the in vivo experiments, we assessed the intrinsic MR properties of the thrombus by measuring the relative signal intensity (SI) compared to the reference adjacent muscle using the formula SI (%) = 100 × [(SI thrombus)/(SI muscle)] on the T_1_-wi and T_2_-wi data. We also analyzed the combined T_1_ × T_2_ signal to assess the discriminatory capacity of KCREKA-NPs. The Kolgomorov–Smirnov normality test with the Dallal–Wilkinson–Lille correction was used to test normality. Non-parametric one-way ANOVA, followed by the Kruskal–Wallis test, was used to compare group means. A value of *p* ≤ 0.05 was considered significant. Statistical analysis was performed using the software GraphPad Prism 9.0.

## 3. Results

### 3.1. Peptide Synthesis

**Figure 1:**
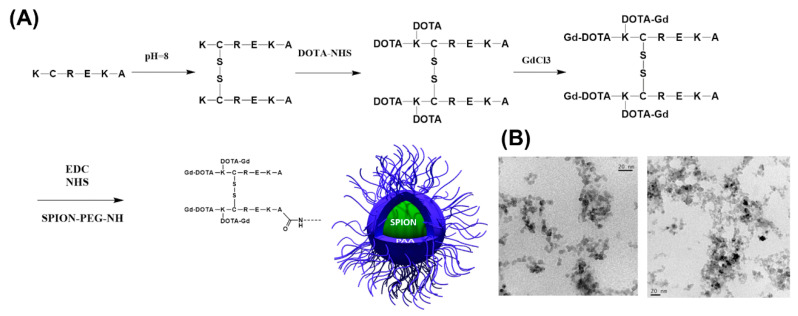
(**A**) Schematic representation of the synthesis of KCREKA-NPs starting from the protected KCREKA peptide. (**B**) TEM images of the KCREKA-NPs.

The general synthetic route is represented in Figure 1A. The peptide with the CREKA sequence was selected based on its known affinity for fibrin [27,36,37], one of the main components of blood clots and the target of fibrinolytic therapy. Since the CREKA sequence is required for fibrin binding, an additional lysine at the N-terminal was included for labeling with Gd in the amine groups. Therefore, the reaction started with the peptide KCREKA with lysine 5 protected with an F-moc group to avoid undesired cross reactions. The molecular weight of the initial peptide was confirmed via MS (MALDI-TOF: *m*/*z* observed: M + H + 980 *m*/*z* calculated: 980). The KCREKA peptide was first oxidized to create a disulfide bridge at the cysteines (m/z observed: M + H + 1941 *m*/*z* calculated: 1941) to reduce the reactivity of the thiol group; then the N-terminal lysine was reacted with three equivalents of DOTA-NHS with the aim of attaching two DOTA groups per KCREKA peptide to a total of four DOTA per dimer (*m*/*z* observed: M + H + 3513 *m*/*z* calculated: 3454–3500), and finally with GdCl_3_ to yield the final Gd-labeled peptide (*m*/*z* observed: M + H + 4054 *m*/*z* calculated: 4082–4032).This strategy for the coupling of the peptide to NP is slightly different to other coupling strategies, where the CREKA peptide is attached to NPs through Cys via thioether links [27,38,39], and instead allows the peptide to be coupled to the nanoparticle’s surface through the carboxylic end group of the peptide.

### 3.2. KCREKA-NP Synthesis

KCREKA-NPs were synthesized by attaching the peptide described above to the PEG chains at the surface of PEG-NPs. The coupling reaction was monitored via HPLC and the conjugation rate of the peptide to the NPs was higher than 95%.

First, we evaluated the effect of the PEG length on the r_1_ and r_2_ relaxivities. PEG-NPs were synthesized with PEGs of 2 kDa, 3 kDa, 6 kDa, and 10 kDa and then DOTA-Gd was attached at the N-termini of the PEGs. Phantom studies showed that the r_2_ relaxivity increased from 120 mM Fe s^−1^ to 170 mM Fe s^−1^ when Gd was attached at the end of the PEG chain, and these values were similar for nanoparticles decorated with 2, 3, and 6 kDa PEG, whereas nanoparticles with a longer PEG chain of 10 kDa showed a decrease in the r_2_ values to 140 mM Fe s^−1^ (Table S1). Regarding r_1_ relaxivity, we observed a slight increase in the r_1_ values depending on the PEG size, achieving a maximum value of 0.69 ± 0.03 mM Fe s^−1^ for PEG-NP with a PEG size of 6 kDa. Based on these results, we selected a 6 kDa PEG coating for the KCREKA-NPs.

**Figure 2:**
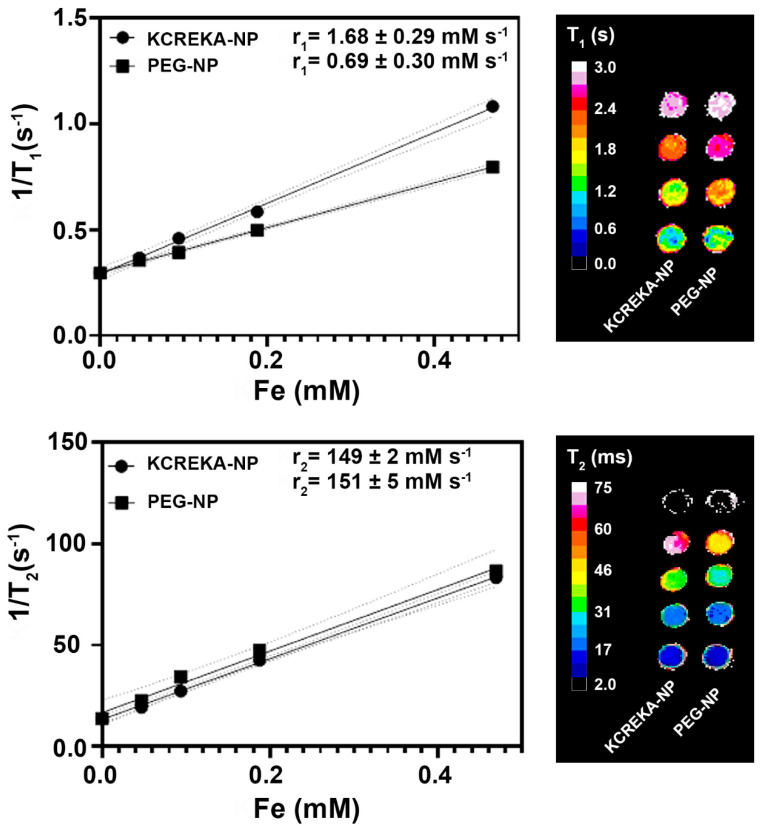
T_1_ and T_2_ map plots vs. Fe concentrations for the calculation of the r_1_ and r_2_ relaxivities of KCREKA-NPs and PEG-NPs. Right images show representations of the phantom for both T_1_ and T_2_ maps.

KCREKA-NPs were synthesized by attaching the peptide to the amino (N) termini of the PEG-NPs. As a control, the same reaction was performed for PEG-NPs but replacing the peptide by Gd-DOTA-NHS, allowing the attachment of Gd to the nanoparticles. The resulting nanoparticles showed a spherical shape in the TEM images (Figure 1B), and the sizes of the nanoparticles, as measured by DLS, were similar for both KCREKA-NPs and PEG-NPs (23 ± 4 nm, PDI = 0.11 and 25 ± 8 nm, PDI = 0.24, respectively). The longitudinal (r_1_) and transverse (r_2_) magnetic relaxivities of both nanoparticles were evaluated in a 9.4 T MRI scanner (Figure 2). The values obtained for r_2_ relaxivities were similar for both KCREKA-NPs and PEG-NPs (149 ± 2 mM Fe s^−1^ and 151 ± 5 mM Fe s^−1^, respectively), whereas the r_1_ relaxivity was higher for KCREKA-NPs (1.68 ± 0.29 mM Fe s^−1^) than for PEG-NPs (0.69 ± 0.3 mM Fe s^−1^). These differences observed in r_1_ values can be attributed to the higher amount of Gd in KCREKA-NPs compared to PEG-NPs (see Table 1).

### 3.3. In Vitro Studies

#### 3.3.1. In Vitro Clot Lysis by rtPA

**Figure 3:**
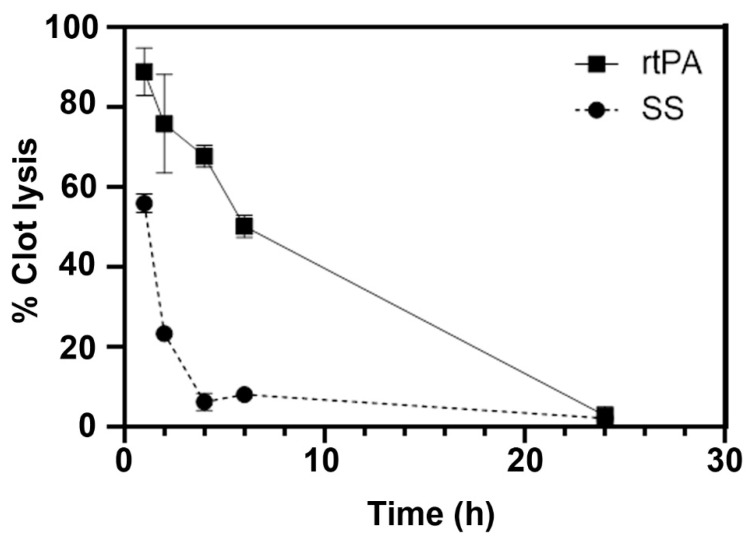
Percentage of in vitro clot lysis upon addition of rtPA or saline solution (SS) at 37 °C.

In vitro blood clots were obtained from fresh blood from control rats. Blood was incubated at 37 °C in direct contact with air. Within 5 min from the start of clotting, fibrin monomers are formed with the cleavage of fibrinogen by thrombin, all of which are present in the blood and triggered by exposure to air. These fibrin monomers then polymerize to create the fibrin structure, which is the main target for the rtPA. However, after the first initial hours of coagulation, the fibers are mechanically stabilized by factor XIII, which protects the newly formed fibrin from fibrinolysis [40]. In our study, we observed that the clot lysing capacity of rtPA decreased over time, with a percentage of clot lysis higher than 70% in the first 4 h that decreased to 50% after 6 h incubation and to less than 5% at 24 h (Figure 3).

#### 3.3.2. In Vitro Clot Imaging

**Figure 4:**
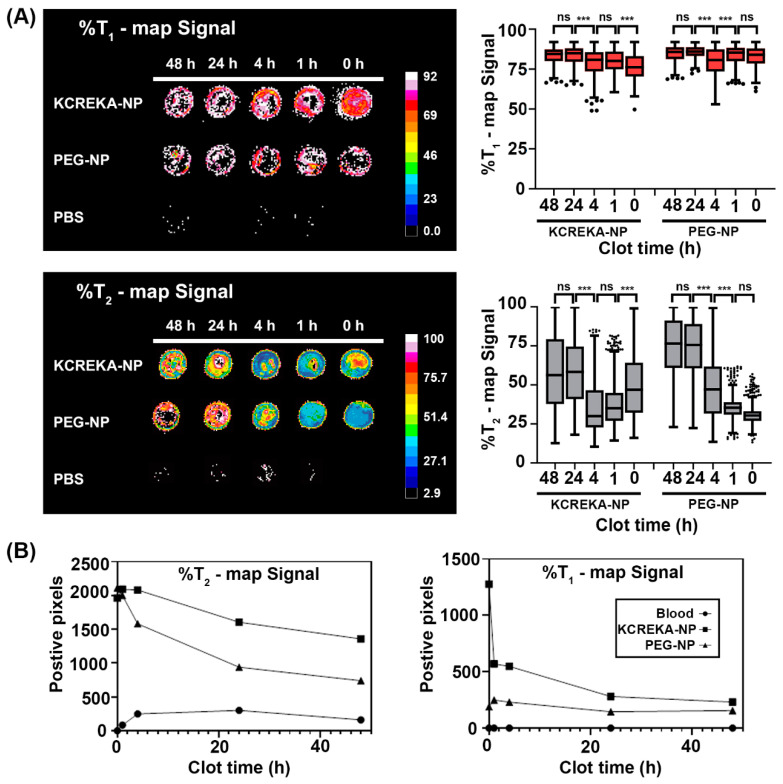
(**A**) MRI images and median values of the decrease in T_1_ and T_2_ weighted images in relation to the background signal for blood clots of 48, 24, 4, 1 h, and 0 h of age incubated with KCREKA-NPs, PEG-NPs, or PBS. (**B**) Number of positive pixels above the background signal for the T_1_ and T_2_ signals for blood clots of 48, 24, 4, 1 h, and 0 h of age incubated with KCREKA-NPs, PEG-NPs, or PBS. *** *p* < 0.001 using one way ANOVA followed by Kruskal-Wallis test.

Blood samples were placed in an agar mold and left for coagulation at 37 °C for 1, 4, 24, and 48 h. After this coagulation step, KCREKA-NPs or control PEG-NPs were added to the clots at the same Fe concentration (0.7 mM Fe_3_O_4_, 2.7 × 10^11^ nanoparticles per clot). As a control (0 h), blood samples treated with EDTA were also incubated for 1 h with both nanoparticles. The clots were then washed several times and placed in an MRI scanner and both T_2_-wi and T_1_-wi were recorded. The minimum values for the agar relaxation rates were used to establish a discriminatory threshold for the background signal noise, and the images were normalized to those threshold values. After applying the background threshold, clots incubated with nanoparticles—both PEG-NPs and KCREKA-NPs—were visible via MRI both in T_1_ and T_2_ maps (Figure 4A), whereas control clots incubated with PBS where almost invisible in the MRI. Therefore, the visible positive pixels could be associated with the presence of nanoparticles in the clot. The number of positive pixels within the clots were higher at low coagulation times and decreased in clots with longer times. In this sense, blood with clotting times less than 4 h showed a higher number of visible pixels than the clots aged for 24 and 48 h. Furthermore, we observed a higher number of positive pixels in the clots for the samples treated with KCREKA-NPs than in samples treated with PEG-NPs (Figure 4B). The higher number of positive pixels in KCREKA-NPs than in PEG-NPs was observed for both T_2_ and T_1_ maps, even though the number of nanoparticles added to the samples were equivalent for KCREKA-NPs and PEG-NPs, suggesting the active targeting of KCREKA-NPs to fresh blood clots.

**Figure 5:**
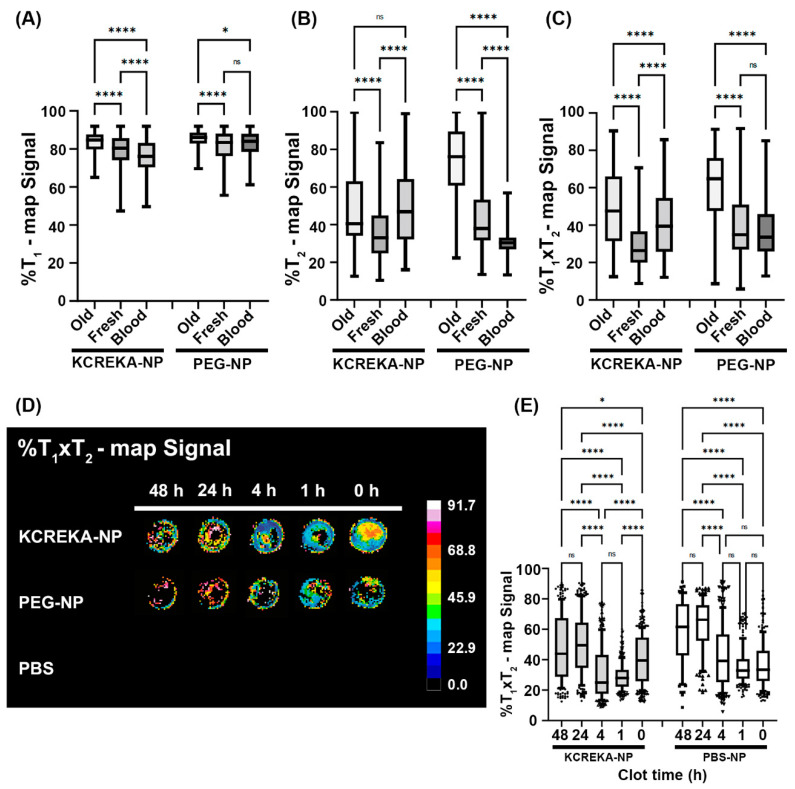
(**A**–**D**) MRI images and median values of the decreases in T_1_, T_2_, and T_1_ × T_2_ weighted images in relation to the background signal for old clots, fresh clots, and blood incubated with KCREKA-NPs, PEG-NPs, or PBS. (**E**) Detailed images of the blood clots with different aging times. * *p* < 0.05, **** *p* < 0.0001 using one way ANOVA followed by Kruskal-Wallis test.

The MR images were analyzed with regard to the percentage of signal reduction from the background, for both T_1_ and T_2_ maps (Figure 5). We studied blood clots at different times after the beginning of the coagulation process. We considered fresh clots to be the samples incubated for 1 and 4 h, which are suitable for thrombolysis with rtPA, as shown previously, whereas samples that had undergone 24 and 48 h of incubation were considered old clots. T_1_ maps showed a significant increase in the signal with the age of the clot, both for KCREKA-NPs and for PEG-NPs. The decrease in signal was more pronounced for fresh clots incubated with KCREKA-NPs compared with old clots (85 [75, 90] vs. 80 [68, 89], *p* < 0.0001); however, the difference between fresh and old clots incubated with PEG-NP was rather small (85 [76, 91] vs. 83 [76, 90], *p* < 0.0001). The T_1_ values measured in control samples were also lower than those in fresh and old clots for KCREKA-NPs, whereas no differences were observed for PEG-NPs (Figure 5A). Regarding the T_2_ maps, we also measured an increase in the MRI signal with the clot age, both for KCREKA-NPs and PEG-NPs (Figure 5B). In this case, the increase with the clot age was higher for blood clots incubated with PEG-NPs than for KCREKA-NPs. Old clots incubated with PEG-NPs showed the lowest MRI signal, decreasing to 76% [47, 96], whereas the fresh clots decreased to 38% [24, 74]. The differences between old and fresh clots were less pronounced when incubated with KCREKA-NPs, ranging from 40% [26, 84] to 33% [20, 66]. Although PEG-NPs showed a greater difference between old and fresh clots compared with KCREKA-NPs, this difference could be attributed to the lower accumulation of nanoparticles in the old clots, as reflected by the lower number of visible pixels in the image (875 vs. 650). Moreover, the T_2_ MRI signal measured for control blood samples incubated with PEG-NPs (30% [23, 42]) was lower than that observed for blood clots with small coagulation times. On the other hand, we observed significant differences among the control samples incubated with KCREKA-NPs (47% [25, 74]) compared with fresh clots, which make these nanoparticles more appropriate for the application of identifying clots that may be suitable for fibrinolytic therapy.

Next, we used a combination of the T_1_ and T_2_ signal with the aim of removing false positives and further decreasing the MRI signals. Here, we multiplied the relaxivity values obtained in both T_1_ and T_2_ maps after normalizing the signal by the background values. The results are shown in Figure 5C,D. The T_1_ × T_2_ values measured for clots incubated with KCREKA-NPs showed a sharp decrease from aged clots compared with fresh clots, from 47% [22, 80] to 26% [15, 51], respectively. Furthermore, the control blood showed a higher value of the T_1_ × T_2_ signal (39% [20, 61]), with the blood clots with low coagulation times exhibiting the lowest values, as measured via MRI. Regarding the samples incubated with PEG-NPs, we also measured a decrease in the T_1_ × T_2_ signal for fresh clots compared with aged clots, 65% [29, 82] vs. 35% [21, 71], respectively. However, the T_1_ × T_2_ signal measured for control blood was not different to the signal measured for clots with low coagulation times (33% [19, 58] vs. 35% [21, 71], *p* = 0.078). Figure 5E shows detailed images of the blood clots with different aging times. Higher domains of low T_1_ × T_2_ signals (blue region) can be seen in the blood clots with clotting times of 1 and 4 h, indicating a higher nanoparticle accumulation. In the absence of nanoparticles, clots incubated only with PBS were not visible via MRI using this method.

The synthesized KCREKA-NPs showed a sharp decrease in the MRI signal compared to the background noise for all the blood clots included in the study. The decrease in the MRI signal was more pronounced for fresh clots compared with old clots and control blood samples, enabling the visualization and the identification of fresh blood clots suitable for thrombolytic treatment with rtPA.

#### 3.3.3. In Vivo Imaging Study

**Figure 6:**
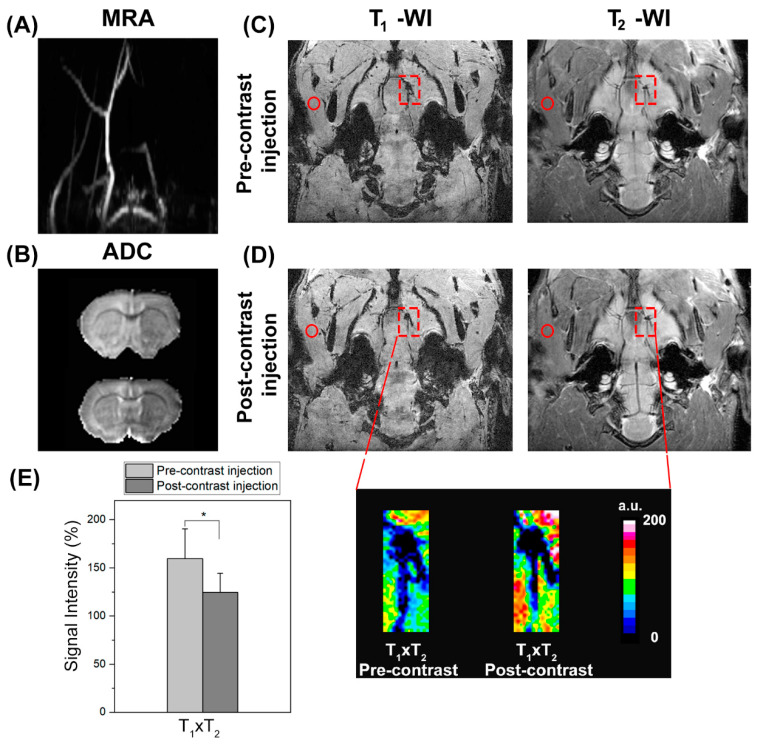
(**A**) Coronal intensity projection of TOF angiography showing right-sided flow deficit of rat brain due to an autologous clot (2 h incubation time). (**B**) Apparent diffusion coefficient (ADC) map at the same level showing a hypointense lesion in the MCA region. Coronal slice from T_1_- and T_2_-wis 3D at the level of the MCA origin pre- (**C**) and post-contrast (**D**) injection of KCREKA-NPs. The squares indicate the thrombus region, and the circles the adjacent muscle were used as reference for signal intensity calculations. (**E**) Corresponding T_1_ × T_2_ expanded reconstruction at the level of the MCA origin, revealing a significant signal intensity change in the presence of the occlusive thrombus after KCREKA-NP injection (* *p* < 0.05).

In vivo contrast signals under open-circulation conditions were assessed in an animal model of thromboembolic stroke. In 21 animals, the thromboembolic stroke model was successfully reproduced, whereas two animals died during surgery. The TOF angiography demonstrated a lack of flow to the right side of the brain in all animals, indicating the presence of the clot (Figure 6A). In the first in vivo experiments, DWI performed before the injection of KCREKA-NPs confirmed an ischemic lesion in all animals imaged with a 34.9% ± 10.9% lesion size relative to the contralateral hemisphere (Figure 6B). Figure 6C,D show coronal T_1_-wis and T_2_-wis taken before and after injection, with the ipsilateral MCA origin and reference adjacent muscle indicated. The corresponding T_1_ × T_2_ expanded reconstruction at the level of the MCA origin revealed a relative signal intensity change in the presence of occlusive thrombus after KCREKA-NP administration. In the clot region, we observed a significant T_1_ × T_2_ signal loss of 24% (159.7% ± 30.7% vs. 121.6% ± 19.8%, *p* < 0.05) (Figure 6E).

**Figure 7:**
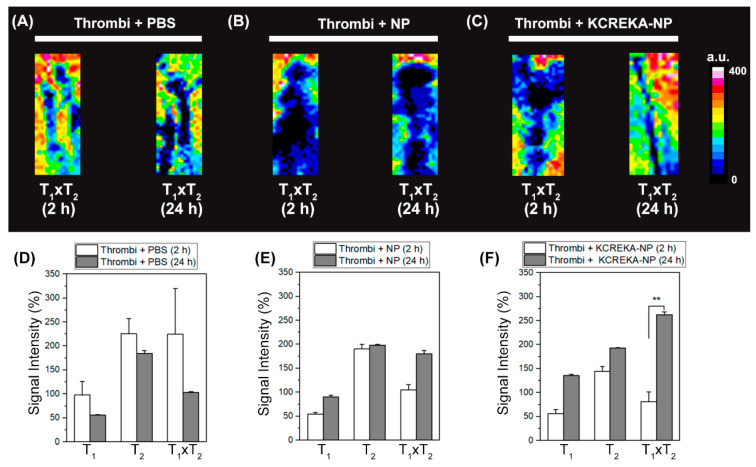
Representative MR T_1_ × T_2_ reconstruction of the thrombus (2 h vs. 24 h incubation time) introduced through the right external carotid artery up to the intracranial internal carotid artery bifurcation to occlude the MCA rat brain. Clots were immersed in (**A**) PBS, (**B**) NP, or (**C**) KCREKA-NP solutions before MCA occlusion in order to assess the signal variation due to the age of the thrombus. Thrombus signal intensity changes (T_1_-wi, T_2_-wi, and T_1_ × T_2_) are plotted over incubation time for the three immersed solutions—(**D**) PBS, (**E**) NP, and (**F**) KCREKA-NP. ** *p* < 0.01, using one way ANOVA followed by Kruskal-Wallis test.

In the second in vivo experiments, clots were incubated for 1 h with either PBS, NPs (untargeted), or KCREKA-NPs, before MCA occlusion. There were no differences in lesion size relative to the contralateral hemisphere among all groups based on DWI. The lesions’ relative sizes were as follows: (i) 41.8% ± 9.8% for PBS, (ii) 47.5% ± 16.2% for NPs, and (iii) 46.1% ± 2.2% for KCREKA-NPs. Figure 7A–C show a representative T_1_ × T_2_ image of fresh clots (2 h formation) and old clots (24 h) incubated for 1 h with nanoparticles. Clots were introduced through the right external carotid artery up to the intracranial internal carotid artery bifurcation to occlude the MCA immediately after 1 h of incubation with the nanoparticles. We quantified the imaging by measuring the SI for the thrombus based on the time and contrast agent used. The relative SI in T_2_-wis was higher than that in T_1_-wis in all groups with a minor change in T_1_, supporting the use of the T_1_ × T_2_ combination for the detection of acute thrombi. Figure 7D allows one to define the thrombus age via MR using the characteristics of T_1_- and T_2_-wis, with results in agreement with previous studies [41]. NP accumulation in the thrombus is readily apparent, indicated by a 41% increase in T_1_ × T_2_ SI (2 h vs. 24 h). Furthermore, the KCREKA-NP probe showed the strongest change—up to 69% (262.5% ± 6.1% vs. 81.3% ± 19.8%, *p* < 0.01)—confirming its capacity to identify fresh blood clots in vivo (Figure 7E,F).

## 4. Discussion

In this study, we developed fibrin-targeted nanoparticles as contrast agents that could help to elucidate whether a blood clot is suitable for recanalization therapy or not. Fibrinolytic therapy with rtPA is the standard pharmacological treatment for ischemic stroke patients [42]. Nonetheless, this treatment has several drawbacks, including the small therapeutic window and low recanalization rates, which make its clinical application difficult [42]. It is well known that the composition of the blood clot strongly affects the activity of the rtPA [14,15]. For instance, cardioembolic strokes caused by blood clots that are rich in fibrin are more likely to be disrupted by rtPA, whereas platelet-rich clots, which are more abundant in atherosclerotic strokes, are less affected by rtPA [17,19]. The current guidelines for treating ischemic stroke recommend proceeding with both intravenous thrombolysis with rtPA and mechanical thrombectomy as soon as possible to remove the blood clot and restore circulation in the brain. Although there are still questions regarding the success of the pre-treatment with rtPA compared with mechanical thrombectomy alone, it has been proven that the administration of rtPA increases the amount of clot that is removed during this procedure [9,10]. Therefore, the nanoparticles developed in this work could be a valuable imaging tool, capable of predicting the effectiveness of rtPA based on the fibrin content of blood clots. On the other hand, recent studies have proven that the fibrin content in blood clots influences the success of recanalization in mechanical thrombectomy [8]. It was observed that the post-procedure serum levels of C-reactive protein were associated with the fibrin content in clots, and therefore could be used to indirectly predict fibrin-rich clots in ischemic stroke patients [8]. Instead, the nanoparticles developed in this work could be used to directly visualize and identify fibrin-rich clots before submitting the patient to recanalization therapy, thus representing a potentially useful tool in both mechanical and pharmaceutical recanalization therapies.

Several fibrin-targeted peptides have been developed for the MRI detection of thrombi, with EP-2104-R (EPIX Pharmaceuticals) being the most advanced one in clinical trials [22,26,43,44]. Fibrin-targeting peptides wearing T_1_ contrast agents showed a 1.5-fold increase in r_1_ values when binding to fibrin, compared to the values measured in buffer or plasma, due to the reduction of the tumbling rate upon protein binding [45]. The fibrin-binding peptide selected for this study was CREKA, a shorter peptide that is widely used for clot imaging, either attached to nanoparticles [27], micelles [37,46], superparamagnetic iron oxide nanoparticles [29], or to Gd chelates [47]. Based on the tumbling effect of peptides on r_1_ values [48], KCREKA-NPs were designed to carry the fibrin-binding peptide CREKA, freely rotating at the nanoparticle surface, through a di-sulfide bond. Upon binding to blood clots, the peptide rotation was slower, resulting in an observed decrease in the T_1_ signal, which was more pronounced in clots with higher fibrin content.

The fibrin content within a blood clot is time-dependent. The coagulation process results in the formation of thrombin, which converts fibrinogen to fibrin, which further polymerizes to form stable blood clots [13]. rtPA binds to fibrin polymers on the clot surface to activate fibrin-bound plasminogen, resulting in the cleavage of fibrin molecules, thus disrupting the blood clot [49]. Thrombolytic resistance to rtPA is often observed in blood clots that are rich in platelets, calcium, cholesterol, and especially excessive fibrin cross-linking polymerization [40,50]. Therefore, there is a need for tools that are capable of elucidating the actual state of the fibrin fibers in blood clots. We observed that the rtPA activity dramatically decreased over time in vitro, with a reduction of 50% after 6 h of clot formation. The decrease in the measured rtPA activity was correlated with the increase in the MRI signal provided by the developed KCREKA-NPs. The in vitro studies showed a sharp decrease in the MRI signals of clots incubated with KCREKA-NPs, which were invisible in the absence of nanoparticles. The decrease in the MRI signal was greater for fresh clots compared with old clots, which could be attributed to the higher binding of KCREKA-NP to fibrin, causing a pronounced decrease in the T_2_ signal, and also to the decrease in the rotation speed of the CREKA peptide upon binding to fibrin, causing the slowing of the molecular rotation of the Gd complex in the peptide [51].

The in vivo studies demonstrated that the main objectives of this work were achieved, with KCREKA-NPs used to visualize blood clots located in the MCA. The KCREKA-NPs administered i.v. were visible through MRI after binding to the blood clot, similarly to other CREKA-targeted nanoparticles [29]. The decrease in the measured T_1_ × T_2_ signal clearly identified the presence of a blood clot in the MCA, which was not visible otherwise; however, this decrease was not sufficient to determine the clot’s age. This could be due to the low injected dose used in this study (1.2 µmol/kg), which was lower than the dose used in the clinical (4 µmol/kg) [26] and preclinical (10 µmol/kg) [52] trials for the EP-2104-R peptide. In a proof-of-concept study, we incubated fresh and old clots directly with KCREKA-NPs and we observed a clear difference in fresh clots compared with old clots in vivo, thus confirming the results obtained in the in vitro study.

The main limitation of this study is related to the in vivo experiments. In this work, we only studied a thromboembolic stroke model, which allowed us to study clots at different aging times. Stroke models induced by platelet-rich thrombi or by the direct injection of thrombin could be interesting models for testing the affinity of the developed KCREKA-NPs.

## 5. Conclusions

In summary, in this study we developed and tested a dual MRI nanoparticle formulation, containing T_1_ and T_2_ contrast agents for MRI analysis. The developed KCREKA-NPs showed affinity for the fibrin content of blood clots, and the MRI signal provided by the nanoparticles showed significant differences depending on the clot age. Although the T_2_ signal could be used to visualize blood clots, discriminating background signals and false positives, the combination of the T_2_ and T_1_ signals (T_1_ × T_2_) allowed us to better differentiate between fresh and old blood clots both in vitro and in vivo. Since fresh clots (less than 6 h from the beginning of coagulation) are more likely to be disrupted by rtPA and to be easily removed via mechanical thrombectomy, the developed KCREKA-NPs could be used as a tool to predict the efficacy of recanalization treatment and improve the triage of ischemic stroke patients.

## Figures and Tables

**Table 1:** Physicochemical properties of KCREKA-NPs and PEG-NPs.

Sample	Core	Gd/ Fe_3_O_4_	Size/nm (Number Average)	Size/nm (Z-Average)	P.I	r_1_/ mM Fe s^−1^	r_2_/ mM Fe s^−1^
KCREKA-NP	Fe_3_O_4_	0.75	23 ± 4	97 ± 2	0.11	1.68 ± 0.29	149 ± 2
PEG-NP	Fe_3_O_4_	0.11	25 ± 8	94 ± 4	0.24	0.69 ± 0.3	151 ± 5

## Data Availability

Statistical analysis plan is available on request.

## Supplementary Materials

The following supporting information can be downloaded at: https://www.mdpi.com/article/10.3390/pharmaceutics14102156/s1, Table S1: Effect of PEG size chain on the physicochemical properties of PEG-NP Figure S1: (A) HPLC chromatograms for the Gd labelled KCREKA peptides and all the intermediate products. (B) HPLC chromatogram of the KCREKA peptide filtrate before and after the coupling reaction with nanoparticles; Figure S2: Protocol diagram summarizing the number of animals included and excluded atnimals per group.
