# Supplementary material for: Fibrin-Targeted Nanoparticles for Finding, Visualizing and Characterizing Blood Clots in Acute Ischemic Stroke
Source: Pharmaceutics. 2022 Oct 10;14(10):2156. doi: 10.3390/pharmaceutics14102156 (PMC9606925; doi:10.3390/pharmaceutics14102156)

Supplementary information for the manuscript:

Fibrin-targeted nanoparticles for finding, visualizing and characterizing blood clots in acute ischemic stroke

María Luz Alonso-Alonso[1], María Pérez-Mato[2], Ana Sampedro-Viana[1], Clara Correa-Paz[3], Paulo Ávila-Gómez[3], Tomas Sobrino[4], Francisco Campos[3], José Castillo[1], Ramón Iglesias-Rey[1*], Pablo Hervella[1*]

[1]Neuroimaging and Biotechnology Laboratory (NOBEL), Clinical Neurosciences Research Laboratory (LINC), Health Research Institute of Santiago de Compostela (IDIS), Santiago de Compostela, Spain
[2]Neurological Sciences and Cerebrovascular Research Laboratory, Department of Neurology and Stroke Center, Neuroscience Area of IdiPAZ Health Research Institute, La Paz University Hospital, Universidad Autónoma de Madrid, Madrid, Spain
[3]Translational Stroke Laboratory (TREAT), Clinical Neurosciences Research Laboratory (LINC), Health Research Institute of Santiago de Compostela (IDIS), Santiago de Compostela, Spain
[4]NeuroAging Laboratory Group (NEURAL), Clinical Neurosciences Research Laboratory (LINC), Health Research Institute of Santiago de Compostela (IDIS), Santiago de Compostela, Spain

Address for correspondence:

Ramón Iglesias-Rey (ramon.iglesias.rey@sergas.es)
Pablo Hervella (pablo.hervella.lorenzo@sergas.es)

Hospital Clínico Universitario, Rúa Travesa da Choupana, s/n 15706 Santiago de Compostela, Spain. Telephone/ Fax number: +34 981951098/+34 981951098

Table S1: Effect of PEG size chain on the physicochemical properties of PEG-NP

**NP Core**	**PEG length (Da)**	**Size**	**P.I**	**r_2 (no Gd) mM Fe s^{-1}**	**r_2 (Gd) mM Fe s^{-1}**	**r_1 (no Gd) mM Fe s^{-1}**	**r_1 (Gd) mM Fe s^{-1}**
Fe3O4	2000	97 ± 2	0.11	123 ± 6	168 ± 6	0.26 ± 0.2	0.58 ± 0.05
Fe3O4	3000	92 ± 6	0.27	120 ± 7	173 ± 7	0.27 ± 0.2	0.65 ± 0.05
Fe3O4	6000	94 ± 2	0.24	102 ± 6	162 ± 7	0.21 ± 0.2	0.69 ± 0.03
Fe3O4	10000	144 ± 4	0.30	96 ± 6	139 ± 6	0.28 ± 0.2	0.62 ± 0.02

Figure S1: (A) HPLC chromatograms for the Gd labelled KCREKA peptides and all the intermediate products.(B) HPLC chromatogram of the KCREKA peptide filtrate before and after the coupling reaction with nanoparticles.

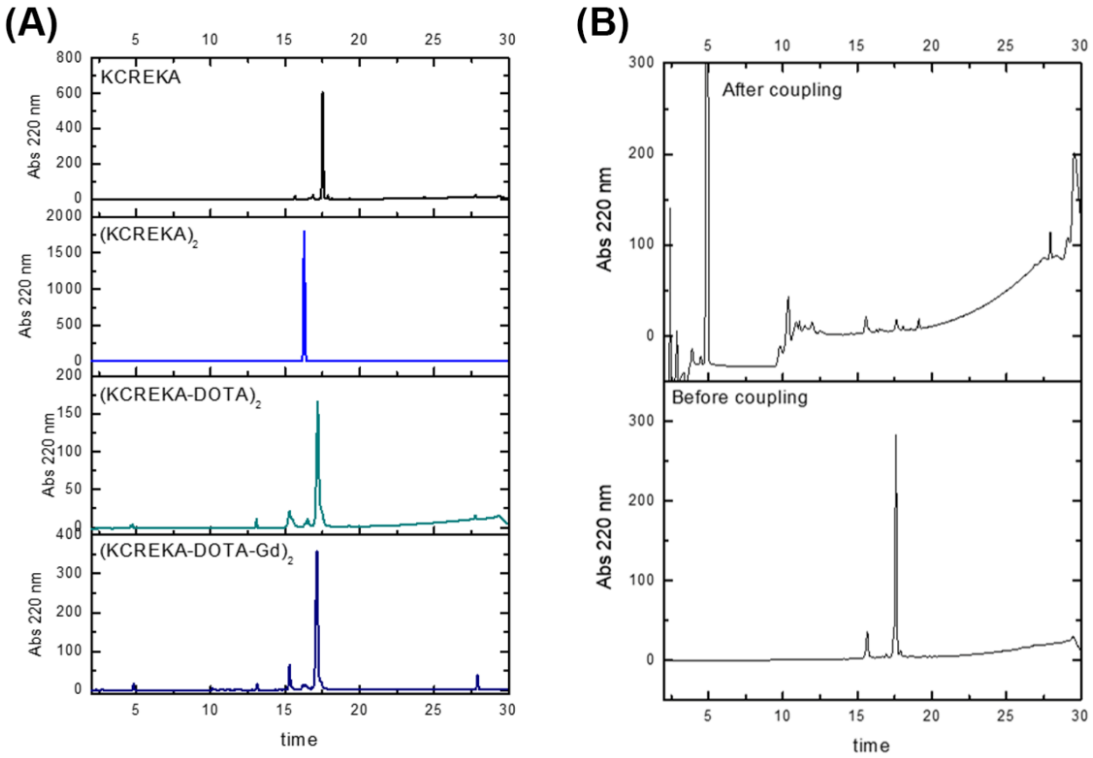

Figure S2: Protocol diagram summarizing the number of animals included and excluded animals per group.

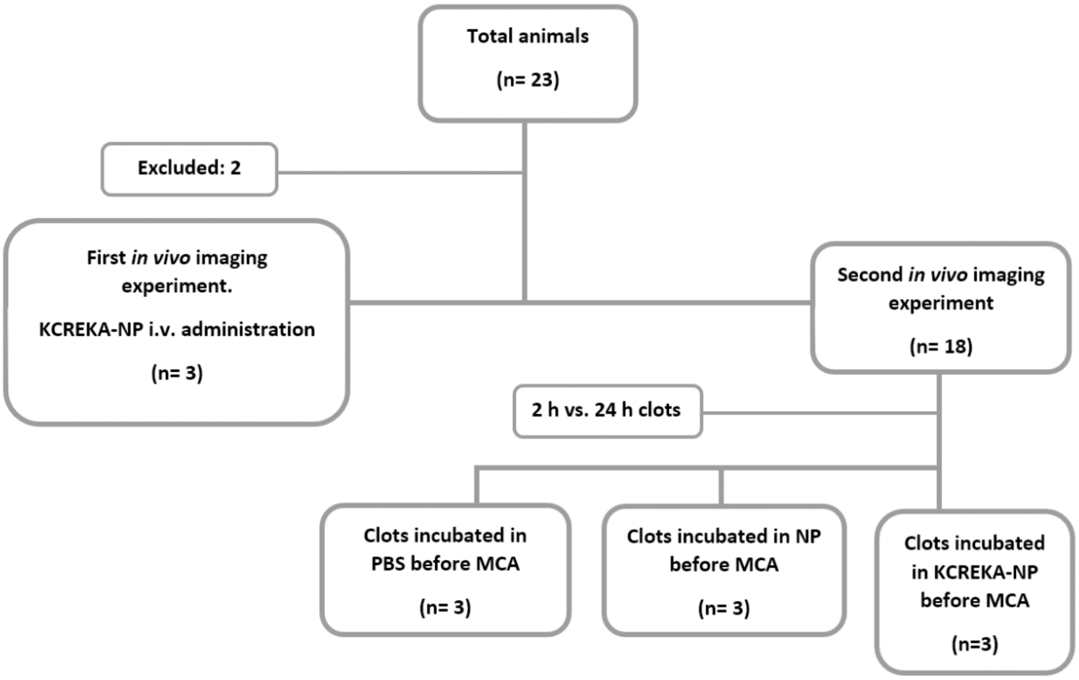

Supplement: Supplementary file 1 [file pharmaceutics-14-02156-s001.zip › pharmaceutics-1938905-supplementary.pdf]
